# Novel Role of Gestational Hydralazine in Limiting Maternal and Dietary Obesity-Related Chronic Kidney Disease

**DOI:** 10.3389/fcell.2021.705263

**Published:** 2021-08-18

**Authors:** Benjamin P. Larkin, Long T. Nguyen, Miao Hou, Sarah J. Glastras, Hui Chen, Rosy Wang, Carol A. Pollock, Sonia Saad

**Affiliations:** ^1^Renal Research Laboratory, Royal North Shore Hospital, Kolling Institute of Medical Research, University of Sydney, Sydney, NSW, Australia; ^2^Department of Cardiology, Children’s Hospital of Soochow University, Suzhou, China; ^3^Department of Diabetes, Endocrinology and Metabolism, Royal North Shore Hospital, Sydney, NSW, Australia; ^4^Faculty of Science, School of Life Sciences, University of Technology Sydney, Sydney, NSW, Australia

**Keywords:** chronic kidney disease, maternal obesity, obesity, fetal programming, DNA methylation

## Abstract

**Background:**

Maternal obesity is a risk factor for chronic kidney disease (CKD) in offspring, underpinning the theory of the developmental origins of health and disease. DNA methylation has been implicated in the programming of adult chronic disease by maternal obesity, therefore, DNA demethylating agents may mitigate offspring risk of disease. In rodent models, low-dose hydralazine has previously been shown to reduce renal fibrosis via DNA demethylation. We used mouse models of maternal obesity and offspring obesity to determine whether administration of low-dose hydralazine during gestation can prevent fetal programming of CKD in offspring.

**Methods:**

Female C57BL/6 mice received high fat diet (HFD) or chow prior to mating, during gestation and lactation. During gestation, dams received subcutaneous hydralazine (5 mg/kg) or saline thrice-weekly. Male offspring weaned to HFD or chow, which continued until endpoint at 32 weeks. Biometric and metabolic parameters, renal global DNA methylation, renal functional and structural changes, and renal markers of fibrosis, inflammation and oxidative stress were assessed at endpoint.

**Results:**

Offspring exposed to maternal obesity or diet-induced obesity had significantly increased renal global DNA methylation, together with other adverse renal effects including albuminuria, glomerulosclerosis, renal fibrosis, and oxidative stress. Offspring exposed to gestational hydralazine had significantly reduced renal global DNA methylation. In obese offspring of obese mothers, gestational hydralazine significantly decreased albuminuria, glomerulosclerosis, and serum creatinine. Obese offspring of hydralazine-treated lean mothers displayed reduced markers of renal fibrosis and oxidative stress.

**Conclusion:**

Gestational hydralazine decreased renal global DNA methylation and exerted renoprotective effects in offspring. This supports a potential therapeutic effect of hydralazine in preventing maternal obesity or dietary obesity-related CKD, through an epigenetic mechanism.

## Introduction

Obesity is a major health problem which predisposes individuals to develop significant consequences, including type 2 diabetes, hypertension, dyslipidaemia, cardiovascular disease, and chronic kidney disease (CKD) (Glastras et al., 2016a). Obesity can cause CKD via mechanisms including alterations in renal hemodynamics, neuroendocrine pathways, promotion of albuminuria, inflammation and oxidative stress (Stenvinkel et al., 2013; Larkin et al., 2018). It is increasingly recognized that the detrimental effects of obesity may be transmitted from one generation to the next, and that maternal obesity has implications for offspring kidney health (Glastras et al., 2018). This phenomenon is in keeping with the theory of the developmental origins of health and disease, which suggests that insults occurring during critical periods of fetal development increase offspring susceptibility to chronic disease later in life (Tain and Hsu, 2017). Using rodent models, we have previously shown that maternal obesity causes renal changes in offspring, such as increased albuminuria, renal fibrosis, oxidative stress and inflammation, all of which promote the development and progression of CKD (Glastras et al., 2016c).

Excess weight and obesity occur in 30–50% of women of reproductive age (Glastras et al., 2018), hence, strategies are needed to minimize the deleterious renal effects of obesity and specifically maternal obesity. CKD in offspring may result from epigenetic changes in the mother that are transmitted to offspring. Hence, therapeutics which influence epigenetic modification of the genome may be useful for CKD prevention. DNA methylation, the most extensively studied epigenetic change, is thought to be heavily involved in the developmental programming of adult chronic diseases by maternal obesity (Cutfield et al., 2007; Sookoian et al., 2013; Nomura et al., 2014). Therefore, drugs with DNA demethylating properties may be beneficial in preventing CKD progression.

Hydralazine, an antihypertensive drug with DNA demethylating actions (Zeisberg and Zeisberg, 2016), has recently been shown to ameliorate renal fibrosis at low doses independently of blood pressure (Tampe et al., 2015, 2017). Given that hydralazine has been used in pregnancy for decades (Kandler et al., 2011), with established safety (Zeisberg and Zeisberg, 2016), we hypothesized that low-dose hydralazine administration to mothers during pregnancy can protect against CKD in offspring due to obesity or maternal obesity. Using mouse models of maternal obesity, offspring obesity and the combination of both conditions, we aimed to demonstrate intergenerational renoprotective effects of hydralazine.

## Materials and Methods

### Animal Experiments

**FIGURE 1:**
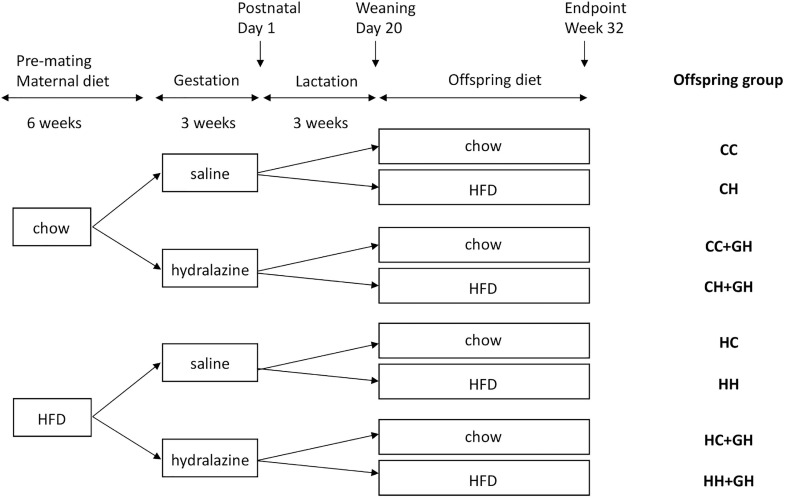
Schematic representation of the animal model used in this study. Mothers continued the allocated diet during gestation and lactation. HFD, high fat diet; CC, control offspring; CH, obese offspring of lean mothers; CC + GH, control offspring exposed to gestational hydralazine; CH + GH, obese offspring of lean mothers with gestational hydralazine; HC, lean offspring of obese mothers; HH, obese offspring of obese mothers; HC + GH, lean offspring of obese mothers with gestational hydralazine; HH + GH, obese offspring of obese mothers with gestational hydralazine.

Ethics approval was granted by the Animal Ethics Committee of the Northern Sydney Local Health District (Resp/17/29), and work complied with the Australian code for the care and use of animals for scientific purposes (National Health And Medical Research Council, 2013). Animal experiments were performed at Kearns Animal Facility of Kolling Institute, Royal North Shore Hospital, Sydney, Australia. Our well-established mouse models of maternal obesity and diet-induced obesity were used in this study (Glastras et al., 2016b). Maternal obesity was induced by feeding 8 week old female C57BL/6 mice high fat diet (HFD 20 kJ/g, 43% of total energy from fat, Specialty Feeds, WA, Australia) for 6 weeks prior to mating and during the gestation and lactation periods. Control dams received standard rodent chow (11 kJ/g, Gordon’s Specialty Stockfeeds, NSW, Australia). Mating commenced at 14 weeks of age, and dams were allowed up to 72 h with males before being returned to their own cages. The presence of a vaginal plug was used to confirm pregnancy. During gestation, mice were injected subcutaneously with either normal saline (0.15 mL) or hydralazine (5 mg/kg) thrice weekly, as this dose is below the blood pressure-lowering dose of 50 mg/kg/day (Tampe et al., 2017). On postnatal day 1, the litter size was reduced to four pups, to avoid milk competition differences. On postnatal day 20, male offspring were weaned to either standard rodent chow or HFD. Only male offspring were studied as previous experimental models of CKD have generally shown that disease progression is more rapid in males than in females (Neugarten and Golestaneh, 2013), and the programming of offspring disease is more frequently observed in male offspring than in females (Cheong et al., 2016a,b). These effects are likely to be related to the differential effects of sex hormones (Whitaker et al., 2012). Offspring continued the same diet, and were weighed fortnightly until sacrifice at 32 weeks of age. There were eight offspring groups with an average of 12 animals per group. The offspring groups are abbreviated such that the first letter refers to the maternal diet (chow or HFD), and the second letter denotes the offspring diet. The groups were: control offspring (CC), lean offspring of obese mothers (HC), obese offspring of lean mothers (CH), obese offspring of obese mothers (HH), control offspring exposed to gestational hydralazine (CC + GH), lean offspring of obese mothers with gestational hydralazine (HC + GH), obese offspring of lean mothers with gestational hydralazine (CH + GH), and obese offspring of obese mothers with gestational hydralazine (HH + GH). A schematic representation of the animal model and experimental groups is shown in Figure 1.

All male offspring underwent intraperitoneal glucose tolerance testing (IPGTT) and blood pressure measurement using a non-invasive CODA^®^ tail vein cuff method (Kent Scientific, CT, United States) during the week prior to the endpoint. For IPGTT, animals were fasted for 5 h, then a glucometer (Accu-Chek^®^, Roche Diagnostics) was used to measure the blood glucose level in tail blood. An intraperitoneal dose of glucose (2 g/kg, Phebra, Australia) was administered, and the blood glucose level was subsequently measured at 15, 30, 60, and 90 min. The trapezoidal rule was used to determine the area under the curve (AUC).

Offspring were harvested at 32 weeks after fasting for 5 h. Isoflurane (2–3%) was used for anesthesia, then cardiac puncture was performed to collect blood. Urine was collected via bladder puncture. The body was perfused with phosphate-buffered saline, prior to harvesting the kidneys, liver and fat. 10% formalin was used to fix tissues for histological analysis; alternatively, they were snapped frozen for extraction of protein, DNA and RNA.

### Serum and Urine Measurements

Insulin was measured in mouse serum (*n* = 5) via the Ultra-Sensitive Mouse Insulin ELISA Kit (Crystal Chem, IL, United States), and insulin resistance index (HOMA-IR) was determined using the following equation: fasting insulin (mU/L) × fasting glucose (mmol/L)/22.5. Serum creatinine was determined (*n* = 8) using a colorimetric assay (Cayman Chemical, MI, United States). Serum was incubated with triacylglycerol reagent and measured colorimetrically to evaluate triglyceride concentration (*n* = 5–6). Non-esterified fatty acids (NEFA) were measured (*n* = 6–10) with a NEFA kit following the manufacturer’s instructions (WAKO, Osaka, Japan).

The spot urine sample was used to calculate the albumin:creatinine ratio (ACR) as a measure of albuminuria. Urine concentrations of albumin and creatinine were determined (*n* = 6–8) using a mouse albumin ELISA kit (Crystal Chem, IL, United States) and a colorimetric assay kit for creatinine (Cayman Chemical, MI, United States).

### Global DNA Methylation Analysis

After extracting genomic DNA from renal tissue (*n* = 4–6) using the ISOLATE II Genomic DNA Kit (Bioline, London, United Kingdom), 5-methylcytosine (5mC) percentage of total DNA was calculated with the Methylated DNA Quantification Kit (Abcam, Cambridge, United Kingdom). An input of 150 ng of genomic DNA was used.

### Renal Structural Changes

To assess glomerulosclerosis, formalin-fixed kidneys (*n* = 6) were sectioned at 2 μm, then underwent Period Acid Schiff (PAS) staining and were evaluated under a light microscope (Leica, Germany). The glomerulosclerosis index was determined for 20 glomeruli per slide by two independent, blinded investigators. Renal cortical collagen I and III deposition were assessed by performing picrosirius red staining on 2 μm kidney sections. Using a digital camera linked to a light microscope (Leica Application Suite, Leica, Germany), four consecutive images were captured and subsequently analyzed by Image J software (National Institutes of Health, United States). Kidneys stained by PAS and picrosirius red were then assessed blindly by two independent investigators for tubulointerstitial fibrosis, tubular vacuolation, tubular dilatation and casts. Representative images of these histological features are shown in Supplementary Figures 1, 2.

### Immunohistochemistry

Formalin-fixed kidneys (*n* = 6) underwent sectioning at 4 μm and were attached to slides. These were deparaffinized, hydrated and heated in a water bath at 99°C for 20 min for antigen retrieval using 0.01 M citrate buffer pH 6. Hydrogen peroxide (Sigma-Aldrich, Dublin, Ireland) was used to quench endogenous peroxidase. Protein Block Serum Free (Dako, Glostrup, Denmark) was used to block slides, which were then incubated overnight at 4°C with primary antibodies against fibronectin (dilution 1:1,000, Abcam, Cambridge, United Kingdom), collagen IV (1:1,000, Abcam), collagen III (1:750, Abcam), collagen I (1:750, Abcam), 8-hydroxydeoxyguanosine (8-OHdG, 1:750, Bioss, MA, United States), and nitrotyrosine (1:500, Merck Millipore, Darmstadt, Germany). Secondary antibody incubation was achieved using the horseradish peroxidase anti-rabbit Envision system (Dako, Japan), and slides were stained with 3,3′-diaminobenzidine tetrahydrochloride (Dako) and Mayer’s hematoxylin. Four to six non-overlapping images per slide were photographed at 200× magnification (Leica Application Suite, Leica, Germany) and the stained area was quantitated by two independent investigators using Image J software.

### Quantitative Real-Time PCR (RT-PCR)

RNA was extracted from kidney tissue (*n* = 6) with the RNeasy Plus Mini Kit (Qiagen, CA, United States), and used to generate cDNA with the iScript cDNA Synthesis Kit (Biorad, CA, United States). The QuantStudio 12K Flex Real-Time PCR System (Thermo Fisher Scientific) was used to perform RT-PCR. Mastermixes incorporated QuantiNova SYBR Green (Qiagen) and the primers listed in Table 1.

**TABLE 1:** Mouse specific primers used in quantitative real-time PCR.

Gene	Forward primer sequence	Reverse primer sequence
18S	ACCGCAGCTAGGAATAATGGA	GCCTCAGTTCCGAAAACC
Catalase	CTCCATCAGGTTTGTTTCTTG	CAACAGGCAAGTTTTTGATG
CD68	CAATTCAGGGTGGAAGAAAG	TCTGATGTAGGTCCTGTTTG
DNMT1	GTGAACAGGAGATGACAAC	CTGGATCCTCCTTTGATTTC
DNMT3a	ACCAGAAGAAGAGAAGAATCC	CAATGATCTCCTTGACCTTAG
Collagen I	CATGTTCAGCTTTGTGGACCT	GCAGCTGACTTCAGGGATGT
Collagen III	TCCCCTGGAATCTGTGAATC	TGAGTCGAATTGGGGAGAAT
Collagen IV	AAGGACTCCAGGGACCAC	CCCACTGAGCCTGTCACAC
Fibronectin	CGGAGAGAGTGCCCCTACTA	CGATATTGGTGAATCGCAGA
MCP-1	GCCTGCTGTTCACAGTTGC	CAGGTGAGTGGGGCGTTA
Nox2	CTACCTAAGATAGCAGTTGATG	TACCAGACAGACTTGAGAATG
Tet3	AGGATCGGTATGGAGAAAAG	CAGGATCAAGATAACAATCACG

### Statistical Analysis

**FIGURE 2:**
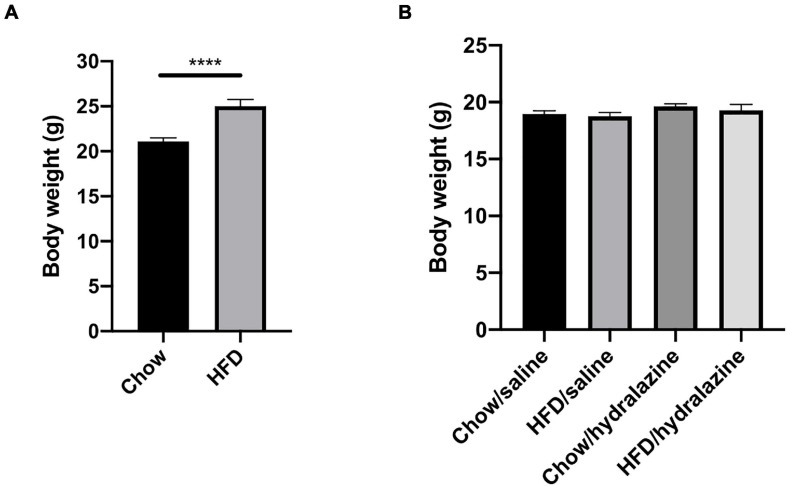
Maternal weight pre-conception and offspring weight at weaning. **(A)** Weight of dams at the commencement of breeding, **(B)** weaning weight of male offspring, according to maternal diet and treatment during gestation. Results expressed as mean ± SEM, ****p < 0.0001.

All results are presented as mean ± SEM. GraphPad Prism version 8.3.1 (San Diego, CA, United States) was used to perform statistical analysis. Analyses were performed using three-way analysis of variance (ANOVA) followed by Fisher’s LSD test. Differences in pre-conception maternal body weight (Figure 2A) were analyzed using an unpaired *t*-test. *P*-values of < 0.05 were considered statistically significant.

## Results

### Biometric Parameters

HFD-fed dams weighed significantly more than those receiving chow (*p* < 0.0001, Figure 2A), confirming the maternal obesity model. There were no pre-conception weight differences between obese dams who later received hydralazine or saline, or lean dams who were later treated with hydralazine or saline. At weaning, body weights of male offspring were similar, regardless of maternal diet or hydralazine treatment (Figure 2B).

At 32 weeks, offspring weaned to HFD weighed significantly more regardless of maternal diet or hydralazine administration (*p* < 0.0001). Body weight was similar between HC and CC (Table 2). While HH weighed significantly more than CC (*p* < 0.0001), their weights were similar to those of CH (Table 2). Gestational hydralazine did not alter offspring body weight at 32 weeks. Kidney weights were increased in CH compared to CC (*p* < 0.01), but were similar between CC, HC, and HH (Table 2). Gestational hydralazine did not affect kidney weights. Kidney:body weight ratio increased in HC compared to CC (*p* < 0.05), and decreased in HH compared to CC (*p* < 0.05, Table 2). CH and CC had similar kidney:body weight ratios. Liver:body weight ratio was similar between CC and HC, but increased above control levels in CH and HH (*p* < 0.0001 for both, Table 2). Gestational hydralazine did not affect offspring liver weight. Epididymal fat:body weight increased in HC, CH, and HH compared to CC (*p* < 0.01, *p* < 0.0001, and *p* < 0.0001, respectively, Table 2). CH and HH had increased retroperitoneal fat:body weight compared with CC (*p* < 0.0001 and *p* < 0.001, respectively, Table 2). Interestingly, retroperitoneal fat:body weight was higher in CH than in HH (*p* < 0.01). Gestational hydralazine did not affect offspring epididymal fat:body weight or retroperitoneal fat:body weight ratios. Mean blood pressure was similar in all offspring groups and remained unaffected by maternal diet, gestational hydralazine and offspring diet (Table 2).

**TABLE 2:** Biometric parameters.

	CC	HC	CH	HH	CC + GH	HC + GH	CH + GH	HH + GH
Body weight (g)	34.65 ± 1.04	33.47 ± 0.67	47.62 ± 1.25 ****,^####^	47.57 ± 0.93 ****,^§§§§^	35.16 ± 0.94	33.70 ± 0.67	48.16 ± 0.71	46.31 ± 1.32
Kidney weight (g)	0.25 ± 0.00	0.29 ± 0.01	0.32 ± 0.02**	0.30 ± 0.01	0.28 ± 0.01	0.33 ± 0.03	0.28 ± 0.02 ϕ	0.32 ± 0.02
Kidney weight/body weight (%)	0.71 ± 0.02	0.87 ± 0.03 *	0.68 ± 0.03^###^	0.62 ± 0.02 *,^§§§§^	0.78 ± 0.03	0.99 ± 0.10	0.59 ± 0.03 ϕ	0.70 ± 0.05
Liver weight (g)	1.39 ± 0.06	1.48 ± 0.06	3.19 ± 0.28 ****,^####^	3.16 ± 0.17 ****,^§§§§^	1.52 ± 0.03	1.59 ± 0.04	3.37 ± 0.11	3.19 ± 0.36
Liver weight/body weight (%)	3.89 ± 0.12	4.41 ± 0.15	6.59 ± 0.49 ****,^####^	6.62 ± 0.31 ****,^§§§§^	4.28 ± 0.15	4.73 ± 0.14	6.99 ± 0.16	6.76 ± 0.60
Epididymal fat weight (g)	0.89 ± 0.11	0.55 ± 0.09 *	2.23 ± 0.11 ****,^####^	2.25 ± 0.12 ****,^§§§§^	0.79 ± 0.09	0.64 ± 0.05	2.05 ± 0.05	1.91 ± 0.13 ^∧^
Epididymal fat weight/body weight (%)	2.53 ± 0.24	1.62 ± 0.25 **	4.66 ± 0.16 ****,^####^	4.72 ± 0.21 ****,^§§§§^	2.18 ± 0.22	1.91 ± 0.15	4.27 ± 0.12	4.27 ± 0.23
Retroperitoneal fat weight (g)	0.34 ± 0.06	0.20 ± 0.07	1.40 ± 0.16 ****,^####^	0.93 ± 0.13 ****,^§§§,‡‡^	0.33 ± 0.04	0.20 ± 0.03	1.47 ± 0.07	0.72 ± 0.12
Retroperitoneal fat weight/body weight (%)	0.81 ± 0.18	0.59 ± 0.19	2.86 ± 0.30 ****,^####^	1.92 ± 0.25 ***,^§§§,‡‡^	0.92 ± 0.09	0.62 ± 0.10	3.04 ± 0.13	1.56 ± 0.26
Mean BP (mmHg)	83.24 ± 2.52	89.98 ± 4.48	82.39 ± 2.20	83.27 ± 3.36	86.11 ± 3.30	95.68 ± 4.11	83.41 ± 2.32	89.86 ± 3.61

*Results expressed as mean ± SEM.*

Compared with CC: *p < 0.05, **p < 0.01, ***p < 0.001, ****p < 0.0001.

*HC vs. CH: ^###^p < 0.001, ^####^p < 0.0001.*

*HC vs. HH: ^§§§^ p < 0.001, ^§§§§^ p < 0.0001.*

*CH vs. HH: ^‡ ‡^p < 0.01.*

*CH vs. CH + GH: ^ϕ^p < 0.05.*

*HH vs. HH + GH: ^∧^p < 0.05.*

### Metabolic Markers

As displayed in Table 3, glucose tolerance was similar between CC and HC. CH and HH both displayed impaired glucose tolerance compared with CC (*p* < 0.0001 and *p* < 0.05, respectively). CH displayed worse glucose tolerance than HH (*p* < 0.001). Gestational hydralazine did not significantly impact offspring glucose tolerance at 32 weeks. Independent of maternal hydralazine treatment, terminal blood glucose level increased in CH and HH compared with CC (*p* < 0.05 and *p* < 0.01, respectively). Terminal blood glucose level was similar between CH and HH. Serum insulin was elevated in CH and HH compared with CC (*p* < 0.05 for both). There were no differences in serum insulin between CC and HC. Gestational hydralazine did not affect serum insulin in offspring. CH and HH had significant insulin resistance compared to CC (*p* < 0.01 and *p* < 0.05, respectively). CC and HC had similar HOMA-IR scores. Gestational hydralazine did not affect insulin resistance in offspring.

**TABLE 3:** Metabolic parameters.

	CC	HC	CH	HH	CC + GH	HC + GH	CH + GH	HH + GH
IPGTT AUC	1,657 ± 66	1,586 ± 77	2,808 ± 231 ****,^####^	2,133 ± 102 *,^§§,‡‡‡^	1,653 ± 109	1,585 ± 59	2,754 ± 144	2,408 ± 93
Glucose (mmol/L)	11.89 ± 0.80	12.65 ± 1.09	14.95 ± 0.92 *	16.37 ± 1.50 **,^§§^	13.27 ± 0.66	11.61 ± 0.72	14.26 ± 0.67	14.19 ± 1.08
Serum insulin (ng/mL)	0.50 ± 0.14	0.25 ± 0.05	2.99 ± 0.91 *,^##^	2.66 ± 0.42 *,^§^	0.40 ± 0.13	0.30 ± 0.06	3.64 ± 1.10	3.75 ± 1.41
HOMA-IR	6.21 ± 1.48	3.41 ± 1.01	52.53 ± 16.28 **,^##^	45.00 ± 9.21 *,^§^	5.27 ± 1.92	3.57 ± 0.94	50.30 ± 10.35	47.16 ± 27.10
Serum triglycerides (mg/mL)	0.49 ± 0.04	0.48 ± 0.07	0.60 ± 0.03	0.73 ± 0.06 **,^§§^	0.58 ± 0.02	0.56 ± 0.07	0.73 ± 0.07	0.52 ± 0.04 ^∧^^∧^
NEFA (mmol/L)	0.62 ± 0.07	0.65 ± 0.05	0.81 ± 0.07	0.90 ± 0.09 *,^§^	0.74 ± 0.10	0.85 ± 0.12	0.92 ± 0.09	0.72 ± 0.10
Urinary ACR (μg/mg)	8.15 ± 2.30	23.89 ± 8.28 *	24.66 ± 4.87 *	41.38 ± 5.76 ****,^§,‡^	11.92 ± 2.67	14.65 ± 3.14	13.85 ± 2.75	18.82 ± 4.69 ^∧^^∧^
Serum creatinine (μmol/L)	81.20 ± 16.12	58.15 ± 16.93	73.07 ± 10.02	185.00 ± 42.12 ***,^§§§§,‡‡‡^	66.83 ± 12.68	69.37 ± 17.19	96.23 ± 20.78	62.67 ± 9.19 ^∧^^∧^^∧^^∧^

*Results expressed as mean ± SEM.*

Compared with CC: *p < 0.05, **p < 0.01, ***p < 0.001, ****p < 0.0001.

*HC vs. CH: ^##^p < 0.01, ^####^p < 0.0001.*

*HC vs. HH: ^§^ p < 0.05, ^§§^ p < 0.01, ^§§§§^ p < 0.0001.*

*CH vs. HH: ^‡^p < 0.05, ^‡ ‡ ‡^p < 0.001.*

*HH vs. HH + GH: ^∧^^∧^p < 0.01, ^∧^^∧^^∧^^∧^p < 0.0001.*

*IPGTT AUC, intraperitoneal glucose tolerance test area under the curve; HOMA-IR, homeostatic model assessment for insulin resistance; NEFA, non-esterified fatty acids; ACR, albumin:creatinine ratio.*

Serum triglycerides were similar between CC, HC, and CH, but were increased above control levels in HH (*p* < 0.01, Table 3). Serum triglycerides were lower in HH + GH than in HH (*p* < 0.01, Table 3). Serum NEFA were similar in CC, HC, and CH, but increased in HH (*p* < 0.05, Table 3). Gestational hydralazine did not alter NEFA in offspring (Table 3).

### Markers of Renal Function/Damage

Urinary ACR increased above CC in HC and CH (*p* < 0.05 for both, Table 3). However, there were no differences between CC + GH and HC + GH (*p* = 0.52), and CC + GH and CH + GH (*p* = 0.62). Urinary ACR also increased in HH compared to CC (*p* < 0.0001, Table 3). As expected, the highest degree of albuminuria was seen in the HH group. Interestingly, HH + GH had reduced urinary ACR compared to HH (*p* < 0.01). Urinary ACR was not improved by gestational hydralazine in CH + GH compared to CH, or in HC + GH compared to HC, and there were no differences between CC + GH, HC + GH, and CH + GH (Table 3).

Serum creatinine was similar between CC, HC, and CH, however it was higher in HH than in CC (*p* < 0.001, Table 3), in keeping with the increased albuminuria seen in this group. Serum creatinine was significantly reduced in HH + GH compared with HH (*p* < 0.0001), which also reflected the findings for albuminuria. No other offspring groups demonstrated any significant benefit on serum creatinine from gestational hydralazine. This suggests that protective effects of hydralazine on offspring serum creatinine are only seen in animals with severe renal impairment due to combined insults of obesity and maternal obesity.

### DNA Methylation

**FIGURE 3:**
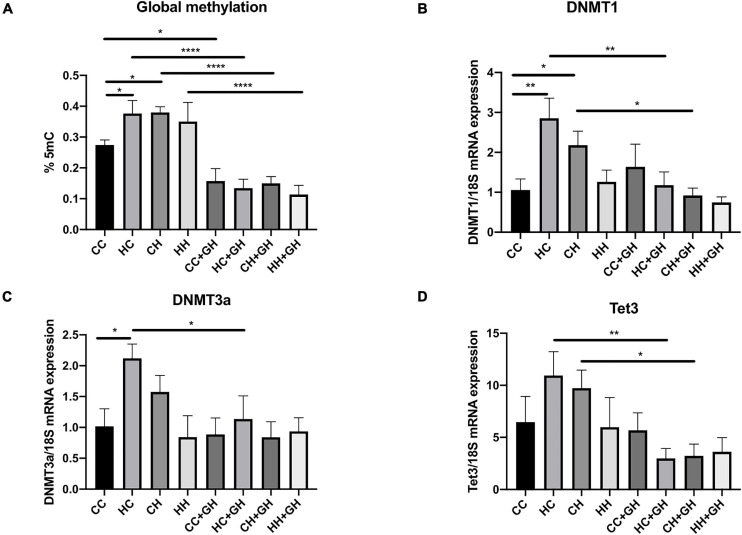
DNA methylation markers. **(A)** Global methylation expressed as % 5 mC of total DNA, **(B)** mRNA expression of DNMT1, **(C)** mRNA expression of DNMT3a, **(D)** mRNA expression of Tet3. Results expressed as mean ± SEM, *n* = 4–6. *p < 0.05, **p < 0.01, ****p < 0.0001. CC, control offspring; HC, lean offspring of obese mothers; CH, obese offspring of lean mothers; HH, obese offspring of obese mothers; CC + GH, control offspring exposed to gestational hydralazine; HC + GH, lean offspring of obese mothers with gestational hydralazine; CH + GH, obese offspring of lean mothers with gestational hydralazine; HH + GH, obese offspring of obese mothers with gestational hydralazine.

Renal global DNA methylation was elevated in HC and CH compared to CC (*p* < 0.05 for both, Figure 3A). In HH, there was a trend toward increased global DNA methylation compared to CC, although this was not significant (*p* = 0.12). In all offspring groups who were exposed to gestational hydralazine, there was a reduction in global DNA methylation. Renal global DNA methylation was reduced in CC + GH compared to CC (*p* < 0.05), HC + GH compared to HC (*p* < 0.0001), CH + GH vs. CH (*p* < 0.0001), and HH + GH compared to HH (*p* < 0.0001, Figure 3A), confirming the efficacy of gestational hydralazine as a DNA demethylating agent.

DNA methyltransferases (DNMT) are key enzymes involved in DNA methylation (Arce et al., 2006), whereas ten-eleven translocation-3 (Tet3) is a DNA demethylating enzyme (Tampe et al., 2017). Renal DNMT1 and DNMT3a mRNA expression increased in HC compared to CC (*p* < 0.01 and *p* < 0.05, respectively), and there was a trend toward higher mRNA expression of Tet3 in HC than in CC (*p* = 0.11, Figures 3B–D). Compared to CC, CH had increased DNMT1 mRNA expression (*p* < 0.05) and trends toward higher mRNA expression of DNMT3a and Tet3 (*p* = 0.19 and *p* = 0.23, respectively, Figures 3B–D). HH had similar mRNA expression of DNMT1, DNMT3a, and Tet3 as controls. Gestational hydralazine reduced DNMT1, DNMT3a, and Tet3 in lean offspring of obese mothers (HC + GH vs. HC, *p* < 0.01, *p* < 0.05, and *p* < 0.01, respectively, Figures 3B–D). Similarly, in obese offspring of lean mothers, gestational hydralazine reduced DNMT1 and Tet3 (CH + GH vs. CH, *p* < 0.05 for both), and there was a trend toward decreased expression of DNMT3a (CH + GH vs. CH, *p* = 0.07, Figures 3B–D). mRNA expression of these enzymes were similar between HH + GH and HH.

### Renal Structural Changes

**FIGURE 4:**
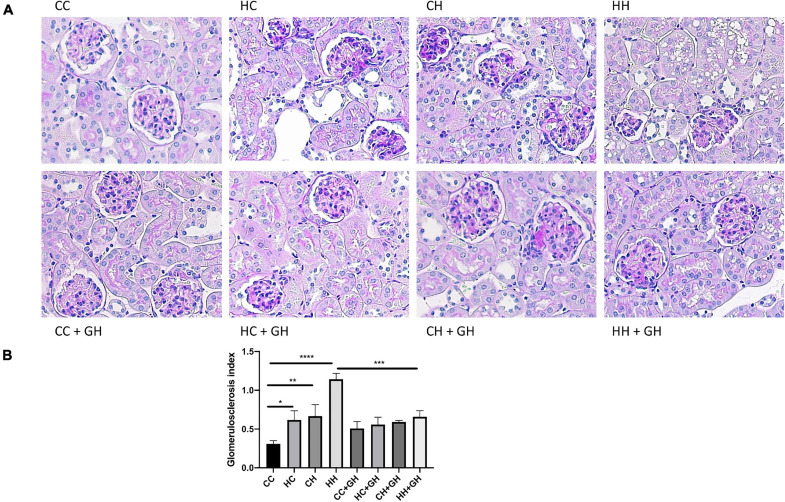
Glomerulosclerosis. **(A)** Representative images of PAS staining for glomerulosclerosis at 400x magnification, **(B)** glomerulosclerosis index. Results expressed as mean ± SEM, *n* = 6. *p < 0.05, **p < 0.01, ***p < 0.001, ****p < 0.0001. CC, control offspring; HC, lean offspring of obese mothers; CH, obese offspring of lean mothers; HH, obese offspring of obese mothers; CC + GH, control offspring exposed to gestational hydralazine; HC + GH, lean offspring of obese mothers with gestational hydralazine; CH + GH, obese offspring of lean mothers with gestational hydralazine; HH + GH, obese offspring of obese mothers with gestational hydralazine.

Reflecting the pattern seen for albuminuria, the highest degree of glomerulosclerosis was observed in HH, where the glomerulosclerosis index (GSI) was approximately fourfold higher compared to CC (*p* < 0.0001, Figures 4A,B). HC and CH also had increased glomerulosclerosis compared to CC (*p* < 0.05 and *p* < 0.01, respectively), and in these groups, the GSI was approximately double that of controls. Gestational hydralazine decreased glomerulosclerosis in HH + GH compared to HH (*p* < 0.001, Figures 4A,B). GSI was not improved by gestational hydralazine in CH + GH compared to CH, or in HC + GH compared to HC and there were no differences between CC + GH, HC + GH, and CH + GH.

**FIGURE 5:**
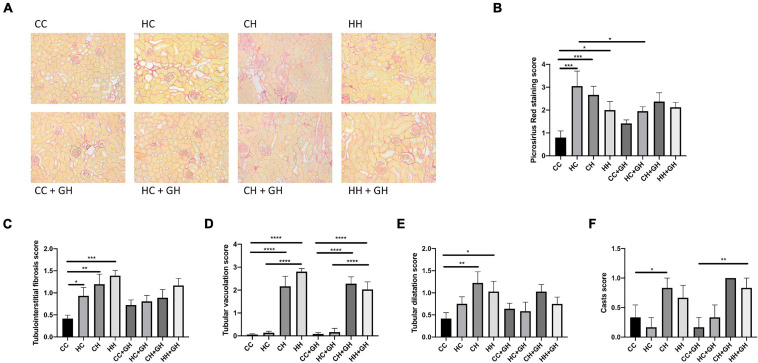
Renal structural changes. **(A)** Representative images of picrosirius red staining at 200× magnification, **(B)** quantitation of picrosirius red staining, **(C)** tubulointerstitial fibrosis, **(D)** tubular vacuolation, **(E)** tubular dilatation, **(F)** casts. Results expressed as mean ± SEM, *n* = 6 *p < 0.05, **p < 0.01, ***p < 0.001, ****p < 0.0001. CC, control offspring; HC, lean offspring of obese mothers; CH, obese offspring of lean mothers; HH, obese offspring of obese mothers; CC + GH, control offspring exposed to gestational hydralazine; HC + GH, lean offspring of obese mothers with gestational hydralazine; CH + GH, obese offspring of lean mothers with gestational hydralazine; HH + GH, obese offspring of obese mothers with gestational hydralazine.

Picrosirius red staining was increased in the kidneys of HC, CH, and HH, compared with CC (*p* < 0.001, *p* < 0.001, and *p* < 0.05, respectively, Figures 5A,B). Gestational hydralazine reduced picrosirius red staining in HC + GH compared to HC (*p* < 0.05), but did not affect staining in other offspring groups (Figures 5A,B).

Increased tubulointerstitial fibrosis was observed in HC, CH, and HH compared to CC (*p* < 0.05, *p* < 0.01, and *p* < 0.001, respectively, Figure 5C). Tubular vacuolation increased above control levels in CH and HH (*p* < 0.0001 for both), but was similar between HC and CC (Figure 5D). Tubular dilatation was higher in CH and HH than in CC (*p* < 0.01 and *p* < 0.05, respectively), but was not significantly increased in HC (Figure 5E). Tubular casts were increased in CH compared to CC (*p* < 0.05), but were similar between CC, HC, and HH (Figure 5F). Gestational hydralazine did not alter tubulointerstitial fibrosis, tubular vacuolation, tubular dilatation, or casts in offspring (Figures 5C–F).

### Renal Fibrosis

**FIGURE 6:**
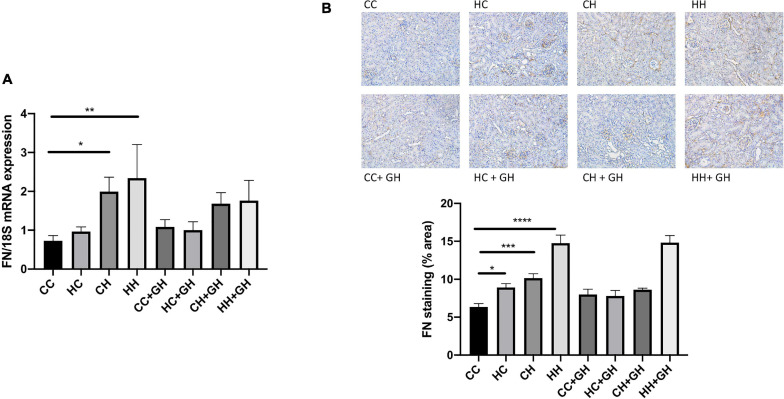
Renal fibrotic changes as measured by fibronectin. **(A)** mRNA expression of fibronectin, **(B)** representative images of fibronectin staining at 200× magnification, and quantitation. Results expressed as mean ± SEM, *n* = 6. *p < 0.05, **p < 0.01, ***p < 0.001, ****p < 0.0001. CC, control offspring; HC, lean offspring of obese mothers; CH, obese offspring of lean mothers; HH, obese offspring of obese mothers; CC + GH, control offspring exposed to gestational hydralazine; HC + GH, lean offspring of obese mothers with gestational hydralazine; CH + GH, obese offspring of lean mothers with gestational hydralazine; HH + GH, obese offspring of obese mothers with gestational hydralazine.

Assessment of fibronectin and collagens I, III and IV was performed to evaluate renal fibrosis. Fibronectin mRNA expression was elevated above control levels in CH and HH (*p* < 0.05 and *p* < 0.01, respectively, Figure 6A). Gestational hydralazine did not attenuate fibronectin mRNA expression in offspring. Renal fibronectin protein expression increased in HC, CH, and HH compared to CC (*p* < 0.05, *p* < 0.001, and *p* < 0.0001, respectively). However, fibronectin protein expression was not significantly upregulated in the HC + GH and CH + GH groups compared to CC + GH, which suggests that gestational hydralazine exerted a protective effect in offspring exposed to either maternal or dietary obesity (Figure 6B).

**FIGURE 7:**
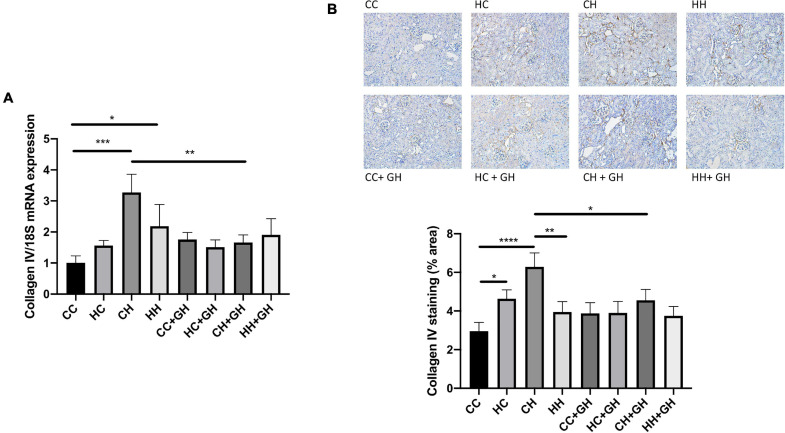
Renal fibrotic changes as measured by collagen IV. **(A)** mRNA expression of collagen IV, **(B)** representative images of collagen IV staining at 200× magnification, and quantitation. Results expressed as mean ± SEM, *n* = 6. *p < 0.05, **p < 0.01, ***p < 0.001, ****p < 0.0001. CC, control offspring; HC, lean offspring of obese mothers; CH, obese offspring of lean mothers; HH, obese offspring of obese mothers; CC + GH, control offspring exposed to gestational hydralazine; HC + GH, lean offspring of obese mothers with gestational hydralazine; CH + GH, obese offspring of lean mothers with gestational hydralazine; HH + GH, obese offspring of obese mothers with gestational hydralazine.

Collagen IV mRNA expression increased in CH and HH compared with CC (*p* < 0.001 and *p* < 0.05, respectively, Figure 7A). Renal collagen IV mRNA expression tended to increase above control levels in HC. There was a trend toward increased collagen IV mRNA expression in CH compared with HH (*p* = 0.06). CH + GH had reduced collagen IV mRNA expression in kidneys compared to CH (*p* < 0.01). However, no protective effects of hydralazine on collagen IV mRNA expression were observed in other groups (Figure 7A). Increased renal collagen IV expression was demonstrated in HC and CH compared to CC (*p* < 0.05 and *p* < 0.0001, respectively, Figure 7B). Collagen IV expression was similar between CC and HH. Interestingly, HH had reduced collagen IV expression compared with CH (*p* < 0.01, Figure 7B). Collagen IV expression was reduced in CH + GH compared to CH (*p* < 0.05), however, gestational hydralazine did not reduce collagen IV expression in any of the other offspring groups.

**FIGURE 8:**
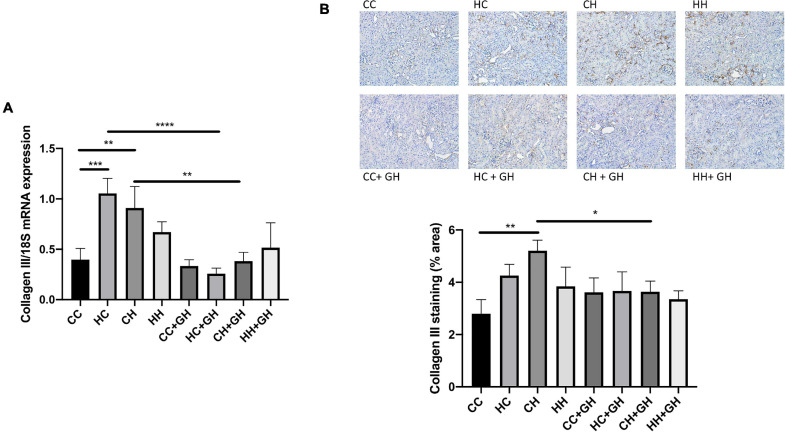
Renal fibrotic changes as measured by collagen III. **(A)** mRNA expression of collagen III, **(B)** representative images of collagen III staining at 200× magnification, and quantitation. Results expressed as mean ± SEM, *n* = 6. *p < 0.05, **p < 0.01, ***p < 0.001, ****p < 0.0001. CC, control offspring; HC, lean offspring of obese mothers; CH, obese offspring of lean mothers; HH, obese offspring of obese mothers; CC + GH, control offspring exposed to gestational hydralazine; HC + GH, lean offspring of obese mothers with gestational hydralazine; CH + GH, obese offspring of lean mothers with gestational hydralazine; HH + GH, obese offspring of obese mothers with gestational hydralazine.

Renal collagen III mRNA expression was elevated above control levels in HC and CH (*p* < 0.001 and *p* < 0.01, respectively, Figure 8A), and there was a trend toward increased expression in HH compared to CC (*p* = 0.17). Gestational hydralazine attenuated collagen III mRNA expression in HC + GH compared to HC (*p* < 0.0001) and in CH + GH compared to CH (*p* < 0.01, Figure 8A). Collagen III protein expression was increased in CH compared to CC (*p* < 0.01, Figure 8B). There was a trend toward increased collagen III expression in HC compared to CC (*p* = 0.06), and expression was similar between CC and HH (Figure 8B). Renal collagen III expression was reduced in CH + GH compared to CH (*p* < 0.05, Figure 8B). In other groups, maternal hydralazine had no beneficial effect on offspring renal collagen III expression.

Renal collagen I mRNA expression was similar in all offspring groups and was unaffected by gestational hydralazine exposure (data not shown). Collagen I protein expression was also similar in all of the offspring groups (data not shown).

### Kidney Inflammation

**FIGURE 9:**
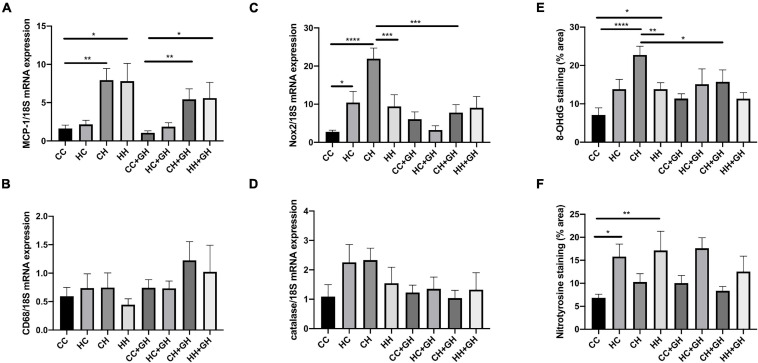
Markers of renal inflammation and oxidative stress. **(A)** Renal MCP-1 mRNA expression, **(B)** renal CD68 mRNA expression, **(C)** renal Nox2 mRNA expression, **(D)** renal mRNA expression of catalase, **(E)** quantitation of renal immunohistochemistry staining for 8-OHdG, **(F)** quantitation of renal immunohistochemistry staining for nitrotyrosine. Results expressed as mean ± SEM, *n* = 6. *p < 0.05, **p < 0.01, ***p < 0.001, ****p < 0.0001. CC, control offspring; HC, lean offspring of obese mothers; CH, obese offspring of lean mothers; HH, obese offspring of obese mothers; CC + GH, control offspring exposed to gestational hydralazine; HC + GH, lean offspring of obese mothers with gestational hydralazine; CH + GH, obese offspring of lean mothers with gestational hydralazine; HH + GH, obese offspring of obese mothers with gestational hydralazine.

Obesity is a known pro-inflammatory state, and as expected, renal monocyte chemoattractant protein-1 (MCP-1) mRNA expression was increased in CH and HH compared to CC (*p* < 0.01 and *p* < 0.05, respectively, Figure 9A). Gestational hydralazine did not affect MCP-1 mRNA expression in offspring kidneys (Figure 9A). Exposure to obesity, maternal obesity or gestational hydralazine did not alter renal CD68 mRNA expression (Figure 9B).

### Renal Oxidative Stress

Obesity and maternal obesity were observed to increase renal oxidative stress. Compared to CC, there was increased renal mRNA NADPH oxidase-2 (Nox2) expression in HC and CH (*p* < 0.05 and *p* < 0.0001, respectively, Figure 9C). There was a trend toward increased Nox2 mRNA expression in HH compared to CC (*p* = 0.07). CH had significantly increased Nox2 mRNA expression compared with HH (*p* < 0.001). Gestational hydralazine attenuated Nox2 mRNA expression in CH + GH compared to CH (*p* < 0.001). There was a near-significant reduction of Nox2 mRNA in HC + GH compared to HC (*p* = 0.05, Figure 9C), however, Nox2 mRNA expression was similar between HH + GH and HH.

Renal catalase mRNA expression was similar between CC, HC, CH, and HH (Figure 9D). Gestational hydralazine did not alter renal catalase mRNA expression (Figure 9D).

8-OHdG renal expression was substantially increased in CH and HH compared to CC (*p* < 0.0001 and *p* < 0.05, respectively), and in CH compared to HH (*p* < 0.01, Figure 9E). Gestational hydralazine attenuated 8-OHdG expression in CH + GH compared to CH (*p* < 0.05), but did not affect expression in other offspring groups (Figure 9E).

Renal immunohistochemistry demonstrated higher nitrotyrosine expression in HC and HH than in CC (*p* < 0.05 and *p* < 0.01, respectively, Figure 9F). Gestational hydralazine did not affect renal nitrotyrosine expression in offspring.

## Discussion

This manuscript uniquely demonstrated that maternal obesity and dietary-induced obesity significantly increased renal global DNA methylation and markers of CKD. Gestational low-dose hydralazine significantly ameliorated albuminuria and glomerulosclerosis due to the combined effects of maternal and offspring obesity, in association with reduced serum creatinine and renal global DNA methylation. Interestingly, hydralazine administration to lean mothers protected obese offspring against renal fibrosis and oxidative stress, suggesting epigenetic regulation *in utero* might have a protective effect on future CKD in offspring.

Increased renal DNMT mRNA expression occurred in the context of maternal obesity and offspring obesity. Consistent with this, elevated DNMT1 expression has been observed in adipocytes of obese humans and mice, in association with insulin resistance through adiponectin promoter hypermethylation and decreased adiponectin mRNA expression (Kim et al., 2015). In obese mouse models, increased DNMT3a expression was observed in adipose tissue, contributing to obesity-related inflammation (Kamei et al., 2010) and insulin resistance (You et al., 2017). Gestational hydralazine administration decreased DNMT mRNA expression in offspring kidneys, and significantly downregulated DNMT1 in lean offspring of obese mothers and in obese offspring of lean mothers. Maternal hydralazine attenuated renal DNMT3a mRNA expression in lean offspring of obese mothers and tended to do so in obese offspring of lean mothers, consistent with previous studies which have shown demethylating effects of hydralazine (Deng et al., 2003; Arce et al., 2006). In our study, there were trends toward increased Tet3 mRNA expression in lean offspring of obese mothers and obese offspring of lean mothers compared to controls. Hydralazine administration during gestation significantly reduced Tet3 mRNA expression in lean offspring of obese mothers, and in obese offspring of lean mothers, compared to relative controls. In murine models of unilateral ureteral obstruction and folic acid nephropathy-induced renal fibrosis, Tampe et al. (2015, 2017) have demonstrated low-dose hydralazine ameliorates renal fibrosis through induction of Tet3-mediated hydroxymethylation, followed by demethylation of the *RASAL1* promoter. This discrepancy likely relates to differences between mouse models, or alterations in other methylation enzymes (Kohli and Zhang, 2013), which could be regulated by hydralazine administration during gestation. The overall effect of gestational hydralazine on global DNA methylation strongly suggests that low-dose hydralazine has a demethylating effect. While global DNA methylation reflects the net effect on gene methylation, it does not show specifically which genes are hyper- and hypomethylated. Although beyond the scope of this study, additional experiments involving whole genome methylation profiling, or assessment of methylation of genes such as *PPARGC1A*, *IGFBP-1*, and *SLC6A4*, which have previously been shown to be associated with T2D and obesity (Ling et al., 2008; Gu et al., 2013; Zhao et al., 2013), may be beneficial.

As previously demonstrated (Glastras et al., 2016b, 2017), HFD-fed offspring developed an obese phenotype with elevated total body weight, kidney, liver, epididymal fat, and retroperitoneal fat weights, independent of maternal diet or hydralazine treatment. At 32 weeks, HFD-fed mice demonstrated adverse metabolic effects including hyperglycemia, glucose intolerance, hyperinsulinemia and insulin resistance, and developed features of CKD including albuminuria, glomerulosclerosis, tubulointerstitial fibrosis, tubular vacuolation, tubular dilatation, in association with renal fibrosis, inflammation, and oxidative stress. Although maternal obesity did not induce features of the metabolic syndrome in offspring, albuminuria, glomerulosclerosis, and tubulointerstitial fibrosis were observed, suggesting a potent effect of maternal obesity in programming offspring CKD. Metabolic and CKD features were significantly potentiated when offspring were exposed to both maternal obesity and diet-induced obesity. This suggests an obesogenic postnatal diet is a more significant contributor to offspring CKD than maternal obesity, consistent with our previous findings (Glastras et al., 2017).

Interestingly, the combination of maternal obesity and diet-induced obesity resulted in less severe changes than in diet-induced obesity alone. Obese offspring of obese mothers had significantly better glucose tolerance, and reduced retroperitoneal fat weight, renal collagen IV, 8-OHdG, and Nox2 mRNA expression than obese offspring of lean mothers. A similar trend was observed for renal mRNA expression of collagen IV. These findings suggest that maternal obesity programs a better adaptation to dietary obesity later in life. This is supported by Monks et al. (2018), who demonstrated that milk from obesity-prone mothers, compared with milk from obesity-resistant mothers, may better protect offspring from the metabolic consequences of an obesogenic diet postnatally.

Albuminuria is a marker of renal damage in several renal diseases, including diabetic kidney disease, hypertensive nephropathy and other primary renal diseases (Lambers Heerspink and Gansevoort, 2015). Its presence implies injury to podocytes resulting in disruption of the glomerular filtration barrier and an inability to maintain selectivity to protein filtration (Cravedi and Remuzzi, 2013). As seen in our study, albuminuria is intrinsically linked with glomerulosclerosis (Peired et al., 2013; Nagata, 2016). Additionally, albuminuria can exert direct toxic effects on proximal tubular cells, leading to tubulointerstitial damage via activation of inflammatory pathways and reactive oxygen species (ROS) generation (Abbate et al., 2006; Mallipattu and He, 2013; Okamura et al., 2013). Therefore, therapeutic approaches to reduce albuminuria represent valid renoprotective strategies (Mallipattu and He, 2013; Lambers Heerspink and Gansevoort, 2015). Our study demonstrated that gestational hydralazine administration does not induce albuminuria and glomerulosclerosis in all of the groups, suggesting a protective effect on the kidney.

In the obese offspring of lean mothers, gestational hydralazine was associated with significant reductions in renal mRNA and protein expression of collagens III and IV, in association with reduced expression of Nox2 and 8-OHdG, independent of albuminuria reduction. Supporting our findings, previous studies demonstrated that hydralazine exerts antioxidant effects by inhibiting NADPH oxidase (Münzel et al., 1996; Daiber et al., 2005).

Obesity is an oxidative stress state (Dludla et al., 2018), and the kidney is highly susceptible to the effects of oxidative stress owing to its high metabolism and vascularity (Small et al., 2012). In kidneys, oxidative stress and inflammation are key factors in initiating cellular damage, which ultimately progresses to renal fibrosis (Ratliff et al., 2016; Glastras et al., 2017). Our data showed that obesity and maternal obesity increased the oxidative stress markers 8-OHdG (a marker of DNA oxidative damage; Sanchez et al., 2018), nitrotyrosine and the NADPH oxidase Nox2, suggesting obesity induces CKD through oxidative stress mechanisms. In animal models of diabetes, increased 8-OHdG was detected in urine and kidney specimens, associated with increased mitochondrial DNA (mtDNA) damage (Kakimoto et al., 2002; Qi et al., 2017). In addition, nitrotyrosine and NADPH oxidases are known to increase mitochondrial ROS production and cause mitochondrial dysfunction (Gao and Mann, 2009; Dikalov, 2011; Teng et al., 2016). It is currently unclear how maternal obesity induces CKD in the offspring, although our data suggest that this can be due to epigenetic changes, oxidative stress and mtDNA damage which is inherited maternally (Sato and Sato, 2013), and the renoprotective effects of hydralazine may occur through these mechanisms. In addition, *in vitro* studies of systemic lupus erythematosus have suggested that hydralazine’s effect on DNA methylation is mediated via the extracellular signal–regulated kinase (ERK) signaling pathway (Deng et al., 2003; Gorelik and Richardson, 2009). How hydralazine administration regulates the ERK pathway in the context of maternal and dietary obesity is currently unknown. It is important to note that the renoprotective benefits of maternal hydralazine occurred independently of blood pressure regulation, given that mean blood pressure was similar in all offspring groups. Intraglomerular hypertension promotes hyperfiltration, damage of the glomerular filtration barrier and ultimately albuminuria (Lin, 2013; Kovesdy et al., 2017), so it was important that differences in blood pressure were removed as possible confounders.

In summary, we observed deleterious renal and metabolic effects of maternal obesity, offspring obesity, and the combination of both conditions. Gestational hydralazine administration to obese mothers attenuated albuminuria, glomerulosclerosis and the elevation of serum creatinine in obese offspring. In addition, gestational hydralazine administration to lean mothers protected against renal fibrosis in offspring who subsequently developed obesity. Further investigation is required to determine the exact renoprotective mechanisms of hydralazine in the subsequent generation, although an epigenetic mechanism is plausible. Our data support the repurposing of hydralazine as a novel strategy during gestation to prevent the development and progression of CKD related to obesity and maternal obesity.

## Data Availability Statement

The original contributions presented in the study are included in the article/Supplementary Material, further inquiries can be directed to the corresponding author/s.

## Ethics Statement

The animal study was reviewed and approved by the Animal Ethics Committee, Northern Sydney Local Health District.

## Author Contributions

BL: conceptualization, data curation, formal analysis, investigation, methodology, writing—original draft, and writing—review and editing. LN: formal analysis, investigation, and writing—review and editing. MH: investigation and writing—review and editing. SG: supervision and writing—review and editing. HC: writing—review and editing. RW: formal analysis and writing—review and editing. CP: conceptualization, funding acquisition, resources, supervision, and writing—review and editing. SS: conceptualization, formal analysis, funding acquisition, methodology, project administration, resources, supervision, validation, and writing—review and editing. All authors contributed to the article and approved the submitted version.

## Conflict of Interest

The authors declare that the research was conducted in the absence of any commercial or financial relationships that could be construed as a potential conflict of interest.

## Publisher’s Note

All claims expressed in this article are solely those of the authors and do not necessarily represent those of their affiliated organizations, or those of the publisher, the editors and the reviewers. Any product that may be evaluated in this article, or claim that may be made by its manufacturer, is not guaranteed or endorsed by the publisher.

## Supplementary Material

Supplementary Figure 1 Renal structural changes. Representative image of PAS staining at 400× magnification demonstrating glomerulosclerosis.

Supplementary Figure 2 Renal structural changes. Representative image of picrosirius red staining of kidney cortex at 200× magnification demonstrating tubulointerstitial fibrosis, tubular vacuolation, tubular dilatation, and casts.
