# Supplementary figures and images for: Novel Role of Gestational Hydralazine in Limiting Maternal and Dietary Obesity-Related Chronic Kidney Disease
Source: Front Cell Dev Biol. 2021 Aug 18;9:705263. doi: 10.3389/fcell.2021.705263 (PMC8416283; doi:10.3389/fcell.2021.705263)

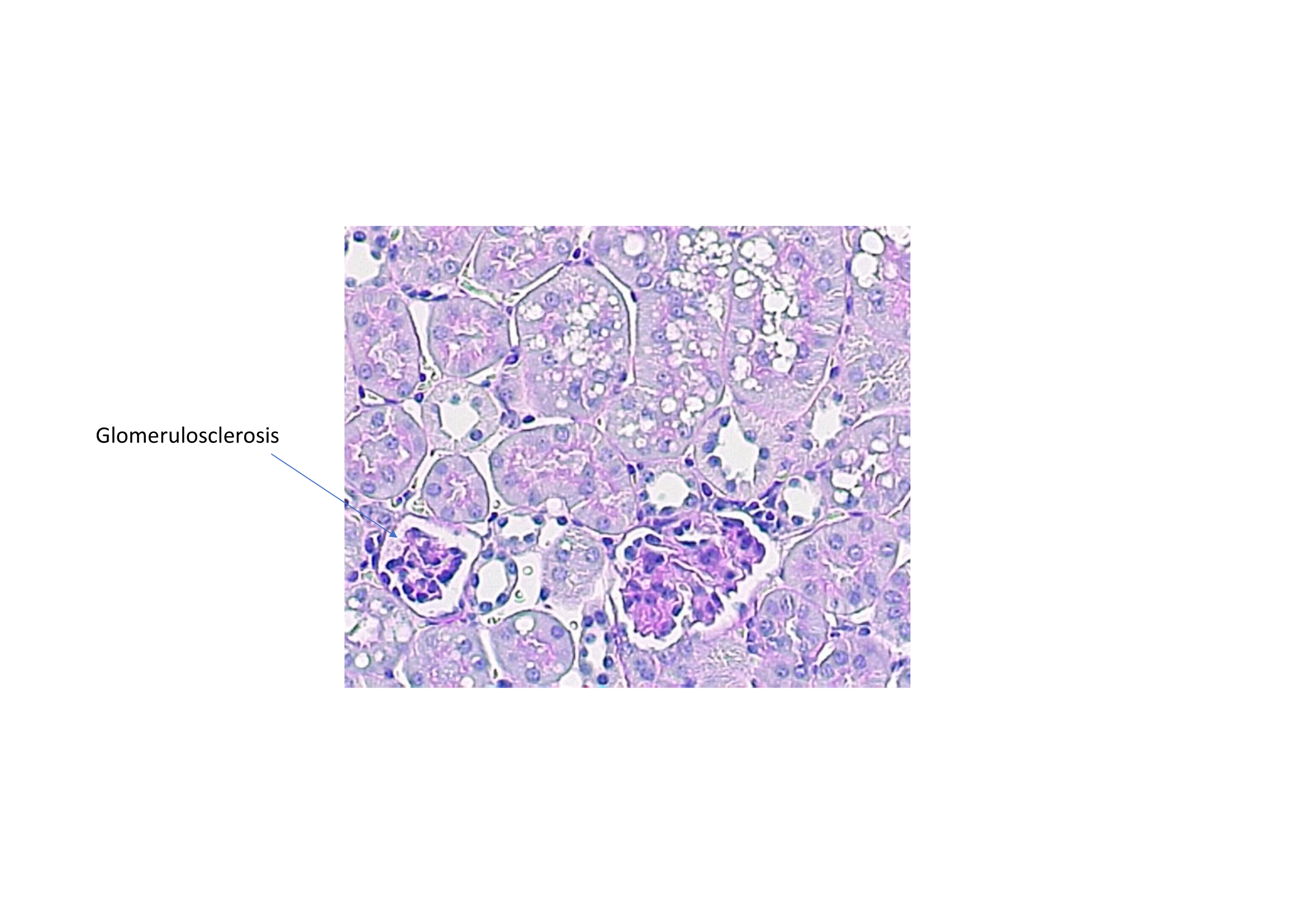

Supplement: Supplementary Figure 1 — Renal structural changes. Representative image of PAS staining at 400× magnification demonstrating glomerulosclerosis. [file Image_1.TIFF]

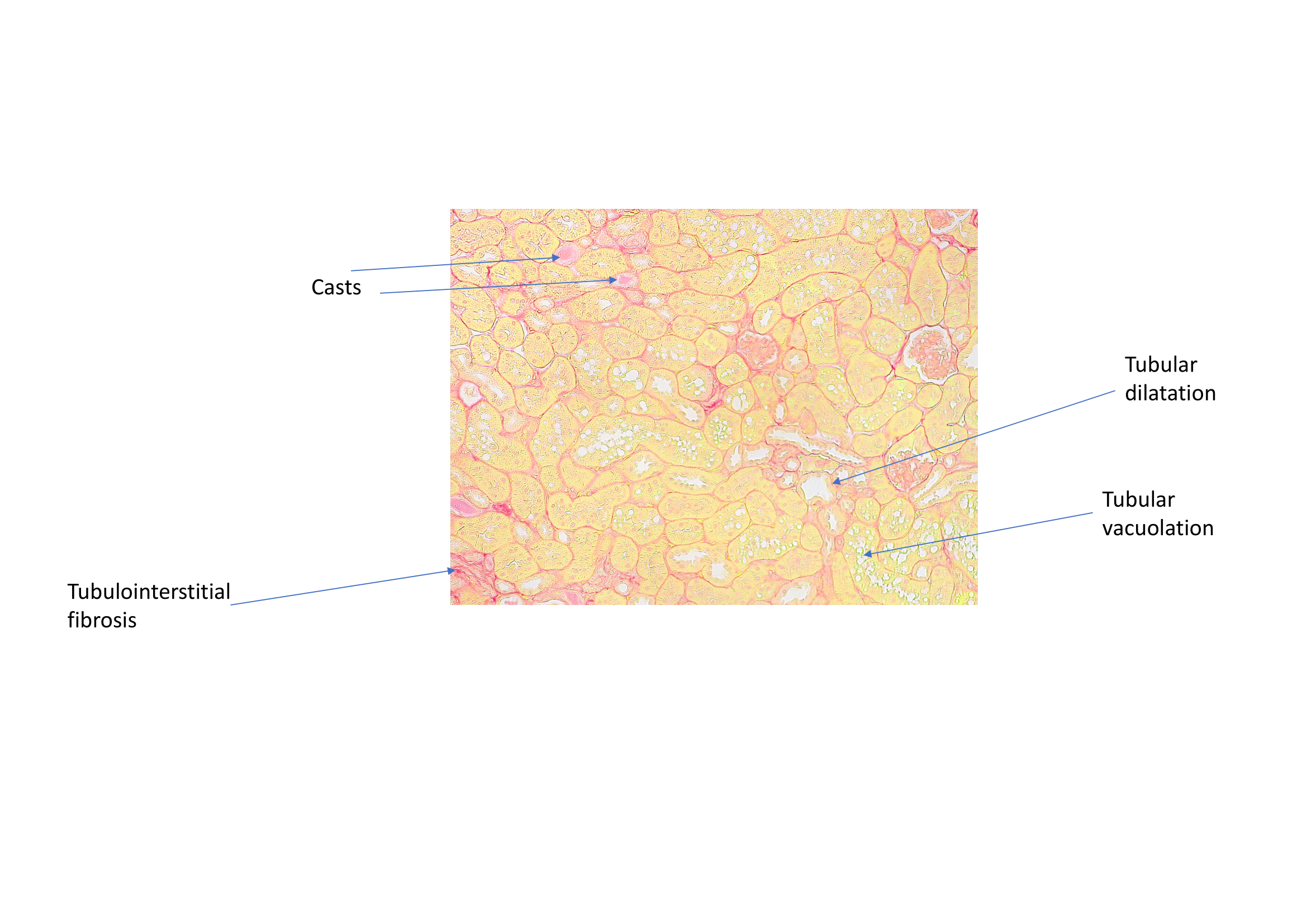

Supplement: Supplementary Figure 2 — Renal structural changes. Representative image of picrosirius red staining of kidney cortex at 200× magnification demonstrating tubulointerstitial fibrosis, tubular vacuolation, tubular dilatation, and casts. [file Image_2.TIFF]
